# Draft genome of *Schizophyllum* sp. strain F4_1A, a novel white-rot fungus isolated in Gauteng Province, South Africa

**DOI:** 10.1128/mra.00830-24

**Published:** 2024-10-31

**Authors:** Grace Nkechinyere Ijoma, Henry Joseph Oduor Ogola, Thendo Mafuna

**Affiliations:** 1 Department of Environmental Science, College of Agricultural and Environmental Sciences, University of South Africa, Roodepoort, Gauteng, South Africa; 2 Department of Biochemistry, University of Johannesburg, Johannesburg, Gauteng, South Africa; University of California Riverside, Riverside, California, USA

**Keywords:** white rot fungus, WGS, Biodegradation, Wood decay

## Abstract

We present a draft genome of *Schizophyllum* sp. F4_1A, a basidiomycete isolated from decaying wood in Gauteng Province, South Africa, sequenced using the MGISEQ-2000RS platform. This draft genome will serve as a valuable genomic resource, enhancing our understanding of white rot fungi and facilitating future comparative genomic analyses.

## ANNOUNCEMENT

*Schizophyllum commune* is a white rot fungi known for producing a diverse range of lignin-modifying enzymes (LMEs) and bioactive compounds, such schizophyllan, highlighting its biotechnological potential (1). Genomic studies have revealed its genetic versatility (2, 3), suggesting diverse phenotypes related to LME production and lignocellulose degradation. Comprehensive genomic analysis across geographic locations is needed to fully understand the genetic plasticity underlying these differences.

**Fig 1:**
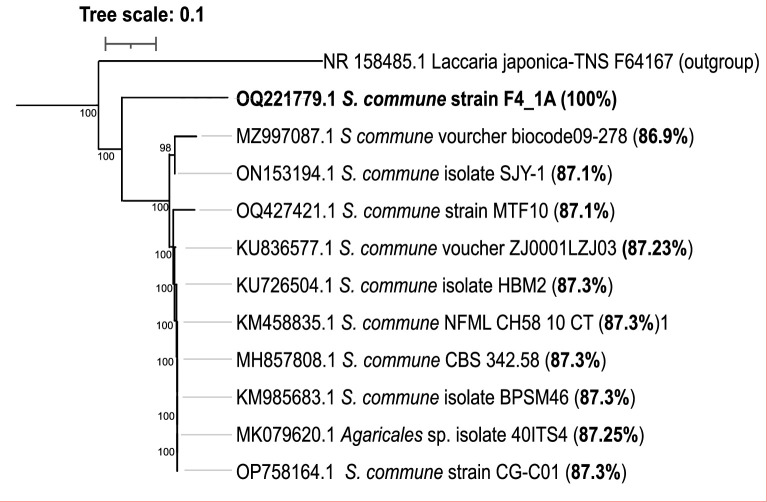
Maximum likelihood phylogenetic tree of *Schizophyllum* sp. strain F4_1A. GenBank numbers are listed in taxon names, and percent homology to strain F4_1A is given in bracket. Numbers at tree nodes represent bootstrap support values (1,000 replications).

We present the genome sequence of *Schizophyllum* sp. strain F4_1A, isolated from decaying wood in Kloofendal Nature Reserve near Johannesburg (4). Spores were extracted from fruiting bodies and cultured on Potato Dextrose Agar (pH 5.5) for 7 days at 25°C in the dark. A monokaryotic strain was selected, and genomic DNA was extracted from a 1 × 1 cm mycelial plug of axenic culture using the Quick-DNA Fungal/Bacteria Miniprep kit (Zymo Research Inc., Irvin, USA), following the manufacturer’s instructions. Phylogenetic analysis of the internal transcribed spacer (ITS1-ITS4) regions revealed less than 87% homology with other *S. commune* species, indicating its distinctness (Fig. 1). Library preparation was performed with an MGI Eazy FS Library Prep Kit and sequenced on a 150 bp paired-end MGISEQ-2000RS platform (MGI Tech, Shenzhen, China) at Agriculture Resource Centre (ARC), Pretoria, South Africa.

A total of 48.4 million reads with an average length of 150 bp and 56% GC content were generated. Quality control was performed using Trimmomatic V0.36 (5) and *de novo* assembly done with SPAdes v3.15.3 (6). The draft genome had 26× coverage, an estimated size of 38,525,979 bp across 1,567 scaffolds, with the longest scaffold being 584,605 bp, and an N50 of 36.9 kb. BUSCO v5.4.2 analysis (7) against the basidiomycota_odb10 data set identified 98.8% genome completeness (93.5% single-copy and 5.3% duplicated BUSCOs).

Gene prediction, performed using Funannotate v1.8.9 (8), involved soft-masking of repetitive sequences using RepeatMasker (9). *Ab initio* gene prediction tools, including Augustus (10), SNAP (11), and GlimmerHMM (12) were used, with *S. commune* H4-8 transcriptome data set (GEO accession: GSE23594) and four *S. commune* protein data sets (NCBI: *GCA_000143185.2*, *GCA_023508725.1*, *GCA_023508785.1*, and *GCF_000143185.2*) supported the predictions. Evidence Modeler (EVM) integrated these sources to produce high-confidence gene models (13). A total of 12,873 genes were annotated, including 12,584 mRNA transcripts and 289 tRNA genes. The average gene length was 1,550.6 bp, and the average protein length was 482.41 amino acids.

Functional annotation using eggNOG-mapper v2.1.12 12 (14) identified 3,854 GO terms, 12,373 COGs, 4,648 KEGG orthologs (KO), 8,716 Pfam domains, 576 CAZymes, and 448 MEROPS peptidases. AntiSMASH v7 (15) detected 62 biosynthetic gene clusters (BGCs) involving NRPS, T1PKS, terpenes, indoles, and phosphonates, with 63 biosynthetic enzymes and 242 secondary metabolism Clusters of Orthologous Groups (smCOGs). SignalP v6.0 (16) identified 951 sequences (7.6%) with signal peptides. Unless otherwise stated, default parameter settings were applied for all software used.

## Data Availability

This genome-sequencing project has been deposited in NCBI GenBank under the BioProject, BioSample, and Sequence reads accession numbers PRJNA107543, SAMN39916750, and SRR27941039, respectively. The *Schizophyllum commune* F4_1A whole-genome shotgun (WGS) project has the project accession JBCLNR000000000. This version of the project (01) has the accession number JBCLNR010000000JBCLNR010000000, consisting of sequences JBCLNR010000001-JBCLNR010001567. The annotation data supporting this study are available on Figshare: https://doi.org/10.6084/m9.figshare.27054694.
